# The Anti-Inflammatory and Antiapoptotic Effects of Nicorandil in Antisepsis Cardiomyopathy

**DOI:** 10.1155/2021/5822920

**Published:** 2021-12-06

**Authors:** Jinshuai Lu, Fen Liu, Xia Yu, Likun Xu, Lingling Zhang

**Affiliations:** ^1^Department of Emergency, People's Hospital of Xinjiang Uygur Autonomous Region, Urumqi, China; ^2^Electrocardiogram Room, Yanggu People's Hospital, Liaocheng, China; ^3^General Practice, People's Hospital of Xinjiang Uygur Autonomous Region, Urumqi, China; ^4^Department of Clinical Laboratory, Hospital of Chengdu University of Traditional Chinese Medicine, Chengdu, China

## Abstract

**Objective:**

To observe the effect of nicorandil on septic rats and explore the possible mechanism of its myocardial protection, so as to provide theoretical basis for the treatment of septic cardiomyopathy.

**Methods:**

Sixty male clean SD rats were selected as the research objects and randomly divided into 3 groups by random number method: sham operation group (sham group), cecal ligation and perforation group (CLP group), nicorandil treatment group (nicorandil+CLP group). After the operation, the nicorandil group was pumped with nicorandil diluent 1 ml/h (2 mg/kg/h) with a micropump for 6 hours. The sham group and CLP group were pumped with the same amount of normal saline 1 ml/h for a total of 6 hours. After 24 hours, the survival of the rats in each group was observed. The expression of troponin I (cTnI), tumor necrosis factor *α* (TNF-*α*), and interleukin-1*β* (IL-1*β*) in the serum was detected. Then, the ventricle was harvested for the observation of the pathological changes of myocardium. Quantitative real-time polymerase chain reaction and immunostaining were used to detect myocardial tissue apoptosis, and Western blot methods were used to detect protein expression changes in nuclear factor-*κ*B (NF-*κ*B) pathways.

**Results:**

24 hours after operation, the survival rate of the rats in the CLP group was 60%. There was a large amount of necrosis of myocardial cells and inflammatory cell infiltration. The survival rate of rats in the nicorandil+CLP group was 75%. Compared with the CLP group, the necrosis of myocardial cells was reduced, and there was still a small amount of inflammatory cell infiltration. In the CLP group, myocardial inflammation and apoptosis were significant, and NF-*κ*B pathway was activated. On the contrary, the NF-*κ*B pathway in the nicorandil+CLP group was inhibited, and the expression of inflammatory factors and apoptosis factors was inhibited.

**Conclusion:**

Nicorandil can reduce the release of inflammatory factors in septic rats, improve the inflammatory response, reduce myocardial damage, and play a myocardial protective effect. Its mechanism may be related to the inhibition of the activation of NF-*κ*B signaling pathway.

## 1. Introduction

Sepsis refers to the systemic inflammatory response caused by infection. Although progress has been made in the treatment of infectious physique, it still occupies the first few deaths in the intensive care unit (ICU). Lipopolysaccharides from Gram-negative bacteria are the main cause of sepsis. Lipopolysaccharides enhance the inflammatory response by activating inflammation-related cells and induce the synthesis and release of related proinflammatory cytokines, and ultimately, the inflammatory response of sepsis may lead to irreversible cardiac dysfunction [1]. The pathophysiology of sepsis includes inflammation, immune dysfunction, and coagulation dysfunction, while sepsis-induced cardiomyopathy is defined as a reversible cardiac dysfunction caused by sepsis [2]. The complex pathogenesis of cardiomyopathy involves the imbalance of inflammatory mediators, mitochondrial dysfunction, oxidative stress, endothelial dysfunction, energy metabolism disorder, and so on. The early infectious agent of sepsis caused by cardiac insufficiency is also a crucial cause of death in sepsis patients [3, 4]. Therefore, it is urgent to treat the dysfunction of sepsis patients effectively.

Nicorandil is an antiangina pectoris drug and is the first ATP-dependent K^+^ channel opener used in clinical practice. Basic studies have shown that nicorandil plays a protective role in a variety of cardiovascular diseases. In rat myocardial infarction models, nicorandil can inhibit PKC activation through opening K^+^-ATP channels, preventing Ca^2+^ overload to reduce ventricular preload and postload, and improve myocardial perfusion volume [5]. Not only that, nicorandil can effectively inhibit apoptosis in diabetes-induced cardiomyopathy and delay the disease process [6]. However, whether nicorandil can delay the course of septic cardiomyopathy and its related mechanisms has not yet been elucidated.

NF-*κ*B is composed of five different DNA-binding proteins and plays a crucial role in the regulation of innate and innate immunity. In physiological state, NF-*κ*B exists in the cell cytoplasm as a polymer. Once stimulated by injury factors, NF-*κ*B immediately dissociates from the complex and forms NF-*κ*B dimer, which enters the nucleus and activates a series of reaction factors such as IL-1*β*, TNF-*α*, and COX2, which are involved in the development and progression of various diseases [7]. In addition, in previous studies, NF-*κ*B has been shown to play a key role in persistent inflammation and has a significant impact on the progression of sepsis cardiomyopathy [8, 9].

Therefore, the purpose of this study was to explore whether nicorandil can improve inflammatory response and apoptosis by inhibiting the NF-*κ*B signaling pathway in CLP-induced cardiomyopathy.

## 2. Materials and Method

### 2.1. Experimental Animals and Groups

60 male SD rats, weighing 330-360 g, were purchased from animal experiment center of Chengdu University of Traditional Chinese Medicine and fed for one week in a day-night cycle of 12 hours at room temperature 18°~ 28°C and relative humidity of 40 ~ 60%. The animal disposal methods in this experiment conform to animal ethical standards. The rats were divided into 3 groups by random number method, namely, sham group, CLP group, and nicorandil+CLP group, with 20 rats in each group. Rats in the sham group underwent cecal exploration without CLP. CLP group and nicorandil+CLP group underwent CLP according to methods reported in the literature to establish sepsis rat models [10]. This study was approved by the Animal Ethics Committee of Chengdu University of Traditional Chinese Medicine Animal Center.

### 2.2. CLP Model

Anesthetized rats were intraperitoneally injected with 1% sodium pentobarbital (3 ml/kg) and fixed on the operating plate to fully expose the abdomen. Then, the skin was incised along the anterior midline of the abdomen for about 2 cm, and the abdominal cavity was opened for exploration and location of the cecum after longitudinal incision along the abdominal white line. Puncture was performed with an 18-point needle to avoid puncturing blood vessels. After removing the needle, a small amount of cecal contents were extruded from the mesenteric perforation hole into the abdominal cavity. Finally, the cecum was returned to the abdominal cavity. After modeling, the nicorandil+CLP group was pumped with 1 ml/h (2 mg/kg/h) of nicorandil diluent (Sigma, USA) by micropump, which lasted for 6 hours. Sham group and CLP group were pumped with 1 ml/h of normal saline, for a total of 6 hours [11].

### 2.3. Measurement of Rat Heart Rate and Arterial Systolic Blood Pressure

After 1% sodium pentobarbital was used to anesthetize the rats, a longitudinal incision was made along the anterior middle of the neck, the right common carotid artery was bluntly separated with hemostats, and the distal end of the right common carotid artery was ligated. Then, cut a small cut below the ligation line, and the heparin-filled epidural anesthesia catheter (Life Technology, Seattle, WA, USA) enters the carotid artery through the small opening, while the other end of the catheter was connected to a computer, and then data was collected.

### 2.4. Specimen Collection

After the model was established, 5 ml blood of the rats in each group was collected from the carotid artery after functional examination. After centrifugation at 3,000 r/min for 10 min, the serum was stored in a refrigerator at -80°C. After blood collection, the chest was opened quickly, and the heart was immediately excised. After removing the atrium and right ventricle, the apex was placed in 4% paraformaldehyde for fixation or stored at -80°C.

### 2.5. Hematoxylin and Eosin (H&E) Staining

The newly taken out rat heart tissues were put into 4% paraformaldehyde and fixed for 24 hours. The fixed specimens were taken out, dehydrated by gradient ethanol, transparent xylene, and embedded in paraffin. After the tissue was cut into thin slices, H&E staining (Jian Cheng, Nanjing, China) was performed, and the histopathological changes were observed under a light microscope. Myocardial histopathological injury score was used to score myocardial edema, interstitial inflammation, and bleeding 24 h after CLP. 0 score for no lesion, 1 score for lesion scope less than 25%, 2 scores for lesion scope 25%-50%, 3 scores for lesion scope 50%-75, and 4 scores for lesion scope greater than 75%. 10 high power fields were observed in each film, and the mean value was used as the pathological injury score.

### 2.6. Immunohistochemical Staining

The sections were blocked with 3% H_2_O_2_ reagent at room temperature for 30 minutes and washed with phosphate-buffered saline (PBS) for 5 minutes. Goat serum blocking solution was added for 30 minutes at room temperature. After shaking off excess liquid, the primary antibody Il-1*β* (Abcam, Cambridge, MA, USA, ab254360) was added at 4°C overnight. The next day, biotinylated secondary antibody (Abcam, Cambridge, MA, USA) was added, and after incubating for 1 hour at room temperature, washed 3 times with PBS for 2 minutes each time. Next, diaminobenzidine (DAB) reagent (Thermo Fisher Scientific, Waltham, MA, USA) was used for color development. After rinsing with distilled water for 1 minute, hematoxylin was used for counterstaining, and hydrochloric acid alcohol was used for differentiation. Finally, after the slices were dehydrated and transparent, they were observed under a light microscope.

### 2.7. Terminal Deoxynucleotidyl Transferase-Mediated dUTP-Biotin Nick End Labeling (TUNEL) Staining

Myocardial tissue sections were taken from each group and performed according to the instructions of TUNEL Kit (Elabscience, Wuhan, China). First, the newly configured TUNEL working solution was used for staining, and the incubation was terminated at 37°C for half an hour. Then, DAPI was used for staining. Positive cells were observed under a fluorescence microscope (Thermo Fisher Scientific, Waltham, MA, USA). The ratio of positive nucleus number to total nucleus number was calculated as the index of apoptosis.

### 2.8. Enzyme-Linked Immunosorbent Assay (ELISA)

The serum of each group of rats was collected, and the content of inflammation-related factors and cardiac troponin was measured by ELISA (Yi Fei Xue, Shanghai, China), including the content of IL-1*β* (YFXESh00018) and TNF-*α* (YFXESh00022) and the content of cTnI (YFXER00200).

### 2.9. Caspase3 and Caspase9 Activity Detection

Took out the myocardial tissue and cut the tissue into small fragments. According to the radioimmunoprecipitation assay (RIPA) instructions (Beyotime, Shanghai, China), an appropriate amount of prepared RIPA lysis solution was added and lysed at 4°C for 30 minutes, centrifuge at 12,000 g at 4°C for 15 minutes, and the supernatant was saved. At the same time, the bicinchoninic acid (BCA) kit (R&D, Minneapolis, MN, USA) was used to detect protein concentration. Then, the activity of Caspase3 (AF835) and Caspase9 (FMK008) in myocardial tissue was determined according to the instructions of the kit method (R&D, Minneapolis, MN, USA).

### 2.10. Western Blot

The extracted protein sample and appropriate loading buffer were heated at 95°C for 5 minutes. Then, 20 *μ*g protein was used for sodium dodecyl sulphate-polyacrylamide gel electrophoresis (SDS-PAGE) electrophoresis, and 280 mA constant flow was transferred to membrane for 1.5 hours. 5% skimmed milk powder was used for blocking for 2 hours. After that, the primary antibody (NF-Kb-p65, Abcam, Cambridge, MA, USA, Rabbit, 1 : 2000, ab32536; Ikk*α*, Abcam, Cambridge, MA, USA, Mouse, 1 : 2000, ab178872; IkB-*α*, Abcam, Cambridge, MA, USA, Rabbit, ab32518; p- IkB-*α*, Abcam, Cambridge, MA, USA, Rabbit, ab133462; *β*-actin, Abcam, Cambridge, MA, USA, Mouse, 1 : 5000, ab8226) was incubated overnight at 4°C. The next day, after washing the membrane with tris-buffered saline-tween (TBST) 3 times, the horseradish peroxidase-conjugated secondary antibody (Abcam, Cambridge, MA, USA) was incubated for 2 hours at room temperature. After washing the membrane three times with TBST, the freshly prepared electrochemiluminescence (ECL) working solution (Gene, China) was added and exposed with a fully automatic imaging analyzer. Image J software was used to analyze the gray value of each band and finally performed the statistical analysis.

### 2.11. RNA Isolation and Quantitative Real-Time Polymerase Chain Reaction (qRT-PCR)

30 mg of rat myocardial tissue was taken; then, 1 ml of TRIzol (Thermo Fisher Scientific, Waltham, MA, USA) was added to it. Shook it and let stand at room temperature for 10 minutes, 1/2 volume of chloroform was added and mixed, centrifuged at 12,000 g for 15 minutes. After aspirating the supernatant, 1/2 volume of isopropanol was added to it, mixed and allowed to stand at room temperature for 10 minutes, and then centrifuged at 12,000 g for 10 minutes, the supernatant was discarded, and the precipitate was removed. 1 ml of 75% ethanol was used to wash RNA precipitation. After drying the pellet in the air, an appropriate amount of RNase-free water was used to dissolve the RNA pellet. An ultraviolet spectrophotometer was used to determine the quality and concentration of RNA samples, and RNA with a ratio of and OD260/OD280 in the range of 1.8 to 2.0 was selected. The complementary deoxyribose nucleic acid (cDNA) first-strand synthesis was carried out according to Vazyme reverse transcription instructions, and the reaction conditions were 85°C, 15 minutes, 55°C, 5 seconds. The cDNA was used as a template and amplified using the Vazyme Real-time PCR kit. The reaction conditions were predenaturation at 95°C for 30 seconds, denaturation at 95°C for 10 seconds, annealing at 60°C for 30 seconds, and 40 cycles. The 2^-*ΔΔ*Ct^ method was used to calculate the relative expression of mRNA. After three independent experiments, statistical analysis was performed. Primers were shown in Table 1.

### 2.12. Statistical Analysis

The results were analyzed statistically using Statistical Product and Service Solutions (SPSS) 22.0 statistical software (IBM, Armonk, NY, USA). Measurement data were expressed as mean ± standard deviation (^−^*X* ± SD), and count data were expressed as percentage. One-way analysis of variance was used for comparison among multiple groups, Least Significant Difference (LSD) test was used for pairwise comparison, and chi-square test was used for comparison of count data. *P* < 0.05 indicates that the difference is statistically significant.

## 3. Results

### 3.1. Nicorandil Improves the Survival Rate and Heart Function of Rats with Septic Cardiomyopathy

**Figure 1:**
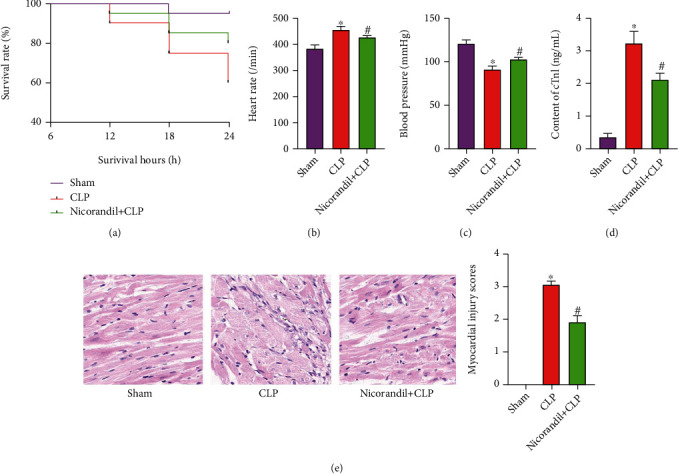
Nicorandil improves the survival rate and heart function of rats with septic cardiomyopathy. (a) The survival rate of rats in all three groups was 24 hours after CLP. (b) Heart rate was detected 24 hours after modeling in three groups of rats. (c) Blood pressure was detected 24 hours after modeling in three groups of rats. (d) The levels of cTnI in serum of three groups of rats. (e) H&E staining of heart tissues in three groups of rats and histological injury scoring system of myocardium. “∗” *P* < 0.05 vs. sham group; “#” *P* < 0.05 vs. CLP group.

This experiment was observed for a total of 24 hours. Among them, 1 animal died in the sham operation group with a survival rate of 95.0%, 8 in the CLP group died with a survival rate of 60.0%, and 5 in the nicorandil+CLP group died with a survival rate of 75.0%. The survival rate of the CLP group was obviously lower than that of the sham operation group, and the survival rate of rats in the nicorandil+CLP group was higher than the CLP group (Figure 1(a)). At the same time, we found that the heart rate of rats in the CLP group was obviously increased 24 hours after modeling, while the heart rate of the rats in the nicorandil+CLP group was obviously lower than that in the CLP group (Figure 1(b)). On the contrary, the blood pressure of rats in the CLP group was obviously reduced, while the blood pressure of rats in the nicorandil+CLP group was obviously increased (Figure 1(c)). The detection of serum cTnI also confirmed that the cTnI content in the CLP group was obviously increased. Compared with the CLP group, the cTnI content in the nicorandil+CLP group was obviously lower (Figure 1(d)). In addition, we used H&E staining to observe the myocardial structure and found that the myocardial cell structure of the sham operation group was normal, and the fibromyoma filament bundles were tightly arranged without obvious edema, degeneration, and necrosis. In the CLP group, a large number of myocardial cells necrosis was seen, showing vacuolar degeneration. The muscle fibers were obviously edema, loosing arrangement, some myocardial fibers were broken, with interstitial edema and inflammatory cell infiltration. Compared with the CLP group, the nicorandil+CLP group still had a small amount of inflammatory cell infiltration, but myocardial cell necrosis was reduced (Figure 1(e)). And the pathological injury score showed that the pathological injury score of the myocardial tissue in the CLP group was 3.21 ± 0.25, significantly higher than that in the Sham group. After nicorandil intervention, the pathological injury score was significantly lower than that in the CLP group (1.89 ± 0.18), and the difference was statistically significant.

### 3.2. Nicorandil Inhibits Inflammation in Rats with Septic Cardiomyopathy

**Figure 2:**
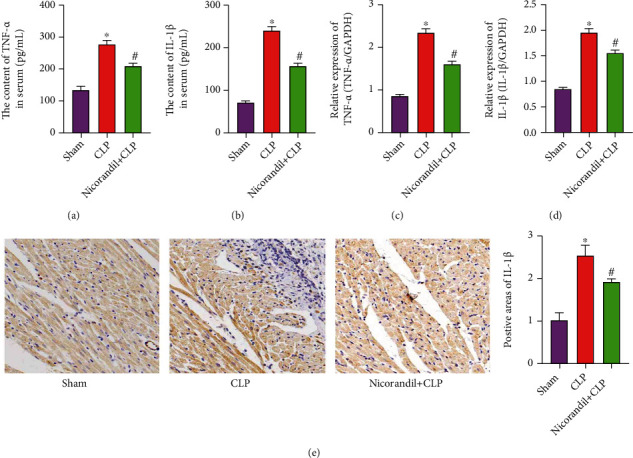
Nicorandil inhibits inflammation in rats with septic cardiomyopathy. (a) The levels of TNF-*α* in serum of three groups of rats. (b) The levels of IL-1*β* in serum of three groups of rats. (c) The mRNA levels of TNF-*α* in myocardial tissue of three groups of rats. (d) The mRNA levels of IL-1*β* in myocardial tissue of three groups of rats. (e) The expression of IL-1*β* in myocardial tissue was detected by immunohistochemical staining and semiquantitative analysis. “∗” *P* < 0.05 vs. sham group; “#” *P* < 0.05 vs. CLP group.

Then, in order to further clarify the influence of sepsis on the degree of inflammation in the rat heart, we detected the content of inflammation-related factors in the serum of each group of rats. The results showed that the levels of IL-1*β* and TNF-*α* in the CLP group were obviously increased, while the levels of these inflammatory factors in the nicorandil+CLP group were lower than those in the CLP group (Figures 2(a) and 2(b)). At the same time, qRT-PCR technology was used to detect the expression levels of IL-1*β* and TNF-*α* mRNA in heart tissue. The results confirmed that the inflammatory response in the heart tissue of the CLP group was significant, while the inflammatory response in the heart tissue of the nicorandil+CLP group was obviously lower than that in the CLP group (Figures 2(c) and 2(d)). In addition, the results of immunohistochemical staining confirmed that the expression of IL-1*β* was obviously increased in the CLP group and obviously decreased in the nicorandil+CLP group (Figure 2(e)).

### 3.3. Nicorandil Inhibits Cardiomyocyte Apoptosis in Rats with Septic Cardiomyopathy

**Figure 3:**
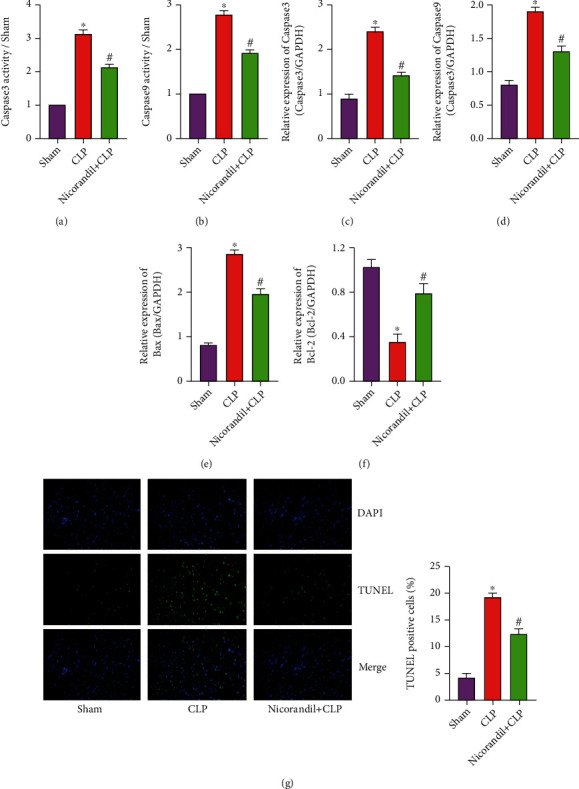
Nicorandil inhibits cardiomyocyte apoptosis in rats with septic cardiomyopathy. (a) The activity of Caspase3 in myocardial tissue of three groups of rats. (b) The activity of Caspase9 in myocardial tissue of three groups of rats. (c) The mRNA levels of Caspase3 in myocardial tissue of three groups of rats. (d) The mRNA levels of Caspase9 in myocardial tissue of three groups of rats. (e) The mRNA levels of Bax in myocardial tissue of three groups of rats. (f) The mRNA levels of Bcl-2 in myocardial tissue of three groups of rats. (g) TUNEL staining was used to detect myocardial apoptosis and statistical analysis. “∗” *P* < 0.05 vs. sham group; “#” *P* < 0.05 vs. CLP group.

When the inflammatory factors in the tissue accumulate excessively, it will trigger an apoptotic response and induce necrosis or normal cells to undergo apoptosis. Therefore, we detected the activity of Caspase3 and Caspase9 in the myocardial tissue of each group of rats, and the results showed that the activity of Caspase3 and Caspase9 in the myocardial tissue of the CLP was obviously increased. In the nicorandil+CLP group, the activities of Caspase3 and Caspase9 were obviously lower than those in the CLP group (Figures 3(a) and 3(b)). qRT-PCR detection of Caspase3, Caspase9, Bax, and Bcl-2 mRNA expression also found that the expression of apoptosis-related factors Caspase3, Caspase9, and Bax increased in the CLP group, while the expression of antiapoptotic factor Bcl-2 was obviously inhibited. On the contrary, in the nicorandil+CLP group, the expression of Caspase3, Caspase9, and Bax decreased, and the expression of Bcl-2 increased obviously (Figures 3(c)–3(f)).

### 3.4. Effect of Nicorandil on the Expression of NF-*κ*B Pathway Related Proteins in Rat Myocardium

**Figure 4:**
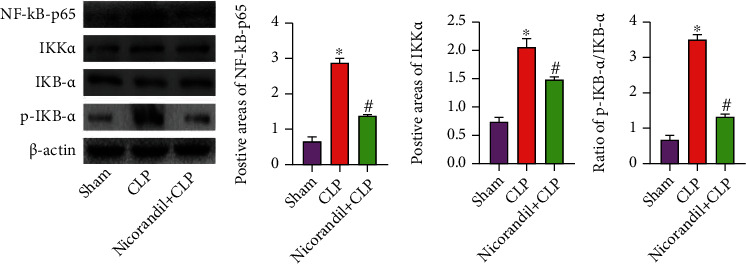
Effect of nicorandil on the expression of NF-*κ*B pathway-related proteins in rat myocardium. Western blot detected the expression of NF-*κ*B, Ikk*α*, IkB-*α*, and p-IkB-*α* in myocardial tissue and analyzed the gray value of the protein bands. “∗” *P* < 0.05 vs. sham group; “#” *P* < 0.05 vs. CLP group.

The WB results showed that the relative gray values of NF-*κ*B and Ikk*α* in the myocardial tissue of the CLP group were obviously higher than that of the sham group, and the nicorandil+CLP group was obviously lower than that of the CLP group. The relative gray value of IkB-*α* in the CLP group was obviously lower than that in the sham group, and the nicorandil+CLP group was obviously higher than that in the CLP group (Figure 4).

## 4. Discussion

Sepsis is a common and frequently-occurring disease in the field of intensive care medicine, and it has become a serious public health burden. Among them, myocardial injury is one of the common organ dysfunctions caused by sepsis, and cardiac dysfunction plays a key role in the clinical outcome of sepsis. The underlying pathophysiology of sepsis cardiac dysfunction is caused by a large number of genetic, molecular, metabolic, and structural mechanisms. These mechanisms are very complex, involving systemic inflammatory system activation, oxidative stress, Ca^2+^ overload, mitochondrial dysfunction, etc. And they may have both independent contributions and highly complex mutual influences [12]. Although many pathophysiological mechanisms have been proposed, the full impact of each mechanism has not yet been elucidated. At present, by measuring biomarkers (cardiac troponin and brain natriuretic peptide) and using echocardiography, it is easy to identify cardiac dysfunction in patients with sepsis [13]. However, there is no specific treatment for septic myocardial depression. Current treatment is still largely supportive [14]. Therefore, future research must focus on targeted therapies aimed at correcting specific pathophysiological abnormalities.

In recent years, nicorandil's research on the treatment of cardiovascular diseases has gradually attracted attention. Nicorandil is a potassium channel opener, which can effectively block the influx of extracellular calcium ions and promote the outflow of potassium ions in the cells, thereby relaxing vascular smooth muscles, dilating blood vessels, and increasing blood flow [15]. A large number of studies have confirmed that nicorandil has a certain protective effect in the treatment of ischemic heart disease and diabetic cardiomyopathy. Nicorandil can effectively reduce inflammation and improve myocardial damage caused by ischemic cardiomyopathy [16]. And nicorandil can inhibit the expression of VCAM-1 in endothelial cells, thereby protecting vascular endothelial cells from damage in diabetic rats [17]. In summary, nicorandil plays a crucial role in the treatment of cardiovascular complications [18]. But its effect on septic cardiomyopathy and its molecular mechanism remains to be studied.

According to relevant literature reports, the best time point to observe myocardial injury in septic rats is 24 hours after the model is created, because the myocardial damage is most obvious [19]. Therefore, this experiment took 24 hours after CLP as the end point of observation. The model evaluation results showed that a large amount of abdominal exudation with foul odor and congestion and necrosis at the end of the cecum was seen 24 hours after the model was opened. At the same time, a large number of myocardial cell necrosis, obvious edema of myocardial fibers, loose arrangement, and inflammatory cell infiltration can be seen under the microscope of myocardial histopathological section, suggesting the successful model of septic cardiomyopathy. cTnI is currently the “gold standard” for detecting myocardial cell damage. It can not only be used as an indicator for the diagnosis of sepsis myocardial injury but also as an indicator of the severity and prognosis of the disease. Therefore, this experiment chose cTnI as the evaluation standard of myocardial damage and its degree after sepsis [20]. Combined with our experimental results, we conclude that nicorandil can reduce the myocardial injury in septic rats.

The NF-*κ*B pathway is involved in cell inflammation, apoptosis, and other links and plays a basic and core role in metabolic inflammation. Under normal circumstances, Rel/NF-*κ*B protein and IKB protein bind to each other, making Rel/NF-*κ*B protein in a state of inhibition. Under the stimulation of hypoxia and other factors, IKK phosphorylates the serine residue of IKB to induce IKB to be degraded by the proteasome, and the released ReIA/p50, ReIA/ReIA, or c-Rel/p50 are translocated to the nucleus, thereby regulating the transcription of target genes and the expression of proteins involved in inflammation, cell growth, and cell survival [21, 22]. Our study confirmed that the NF-*κ*B pathway was activated in the CLP group, the expression of NF-*κ*B-p65, Ikk*α*, and p-IkB-*α* was upregulated, while the expression of the inhibitory protein IkB-*α* was downregulated. Combined with the increase of inflammatory factors and apoptosis in septic cardiomyopathy, it indicates that the NF-*κ*B pathway was activated in CLP rat myocardium, and the effects of nicorandil on inflammation and apoptosis may be mediated by this pathway. Furthermore, our results further update the protective effect of nicorandil beyond acute lung injury [23].

NF-*κ*B can induce the expression of a large number of proinflammatory factors, such as TNF-*α* and IL-1*β*, and these proinflammatory cytokines can directly cause host damage. Studies have found that TNF-*α* and IL-1*β* are the main mediators of cardiac insufficiency in sepsis and are considered to be direct myocardial depressants [24]. In our study, nicorandil downregulated the expression of TNF-*α* and IL-1*β* and reduced inflammation in myocardial tissue, thereby improving cardiac function to a certain extent in rats. In addition, Caspase family and Bcl family are common apoptosis control genes [25]. Caspase is the core of cell apoptosis and a key protein family that performs cell apoptosis. In the Bcl family, whether cells undergo apoptosis is mainly determined by the expression of Bcl-2 and Bax proteins. In this study, compared with the CLP group, the apoptosis of cardiomyocytes in rats treated with nicorandil was significantly inhibited, confirming that nicorandil can improve the apoptosis of cardiomyocytes after CLP to a certain extent, thus, improving some cardiac functions of rats.

## 5. Conclusions

Nicorandil can reduce the release of inflammatory factors in septic rats, improve the inflammatory response, reduce myocardial damage, and thus exert myocardial protection. Its mechanism may be related to the inhibition of the activation of NF-*κ*B signaling pathway by nicorandil.

## Figures and Tables

**Table 1:** Real-time PCR primers.

Gene name	Forward (5′ > 3′)	Reverse (5′ > 3′)
Bax	CAGTTGAAGTTGCCATCAGC	CAGTTGAAGTTACCATCAGC
Bcl-2	GACTGAGTACCTGAACCGGCATC	CTGAGCAGCGTCTTCAGAGACA
Caspase3	TGGAACAAATGGACCTGTTGACC	AGGACTCAAATTCTGTTGCCACC
Caspase9	TCCTGGTACATCGAGACCTTG	AAGTCCCTTTCGCAGAAACAG
TNF-*α*	CCTCTCTCTAATCAGCCCTCTG	GAGGACCTGGGAGTAGATGAG
IL-1*β*	GCAACTGTTCCTGAACTCAACT	ATCTTTTGGGGTCCGTCAACT
GAPDH	ACAACTTTGGTATCGTGGAAGG	GCCATCACGCCACAGTTTC

RT-PCR: quantitative reverse-transcription polymerase chain reaction.

## Data Availability

The datasets used and analyzed during the current study are available from the corresponding author on reasonable request.
